# The Beneficial Effect of Resveratrol on the Quality of Frozen-Thawed Boar Sperm

**DOI:** 10.3390/ani13182829

**Published:** 2023-09-06

**Authors:** Kampon Kaeoket, Panida Chanapiwat

**Affiliations:** Semen Laboratory, Department of Clinical Sciences and Public Health, Faculty of Veterinary Science, Mahidol University, Nakhon-Pathom 73170, Thailand; kampon.kae@mahidol.edu

**Keywords:** antioxidant, boar, frozen-thawed sperm, lipid peroxidation, resveratrol

## Abstract

**Simple Summary:**

Cryopreservation protocol induces reactive oxygen species formation and leads to a decrease in post-thawed sperm quality and fertilizing capacity. We investigated the effect of resveratrol and its optimal concentration on the quality of post-thawed boar semen and the level of malondialdehyde (MDA). Our investigations showed that the supplement of 50–100 µM resveratrol enhanced the quality of the post-thawed semen and decreased the MDA level. However, the maximum concentration of resveratrol (250 µM) tended to decrease sperm motility, sperm viability, intact acrosomes, and mitochondrial membrane potential. The protective or cytotoxic effect of resveratrol on the quality of frozen boar sperm depends on the concentration of resveratrol and extender composition as well as limiting antioxidant uptake capacity in sperm.

**Abstract:**

This study aimed to determine the effect of resveratrol and its optimal concentration on the quality of frozen-thawed (FT) boar sperm. Semen ejaculates were obtained from 13 Duroc boars aged between 1.5 and 3 years. The sperm sample was separated into 7 groups based on the concentrations of resveratrol in the freezing extender, which were 0 (control), 25, 50, 75, 100, 125, and 250 µM, respectively. The sperm was frozen using liquid nitrogen vapor and thawed at 50 °C for 12 s. After thawing, total motility, progressive motility, viability, intact acrosomes, mitochondrial membrane potential and level of MDA were assessed. The supplementation of 50–100 µM resveratrol improved the sperm motility and viability of FT sperm in comparison to the control group (*p* < 0.05). Furthermore, the 50 µM resveratrol group was significantly more protective than the control group in terms of intact acrosome, mitochondrial membrane potential, and level of MDA (*p* < 0.05). Nonetheless, the detrimental effect of resveratrol was found at a concentration of 250 µM. In conclusion, the addition of 50–100 µM resveratrol to a freezing extender is the optimal concentration for enhancing the quality of cryopreserved boar sperm.

## 1. Introduction

Sperm cryopreservation is a valuable technology for the long-term preservation of male fertility in humans and domestic animals. The cryopreservation procedure induces sperm damage in a number of ways under the impact of multiple factors, including significant fluctuations in temperatures, osmotic imbalances, and oxidative stress [1]. Oxidative stress is one of the factors responsible for sperm injury during sperm cryopreservation. During the cooling and freezing procedure, the sperm is markedly exposed to oxidative stress. The cryopreservation protocol induces reactive oxygen species (ROS) formation and ROS assault on sperm, resulting in a decrease in sperm quality and fertilizing capacity [2,3]. Boar spermatozoa are extremely sensitive to oxidative damage by ROS due to the abundance of polyunsaturated fatty acids (PUFAs) in plasma membranes and its limited antioxidant system in the cytoplasm [4]. To mitigate the negative effects of reactive oxygen species on sperm performance, antioxidant compounds have been introduced to the freezing extender to combat or prevent oxidative stress. In the last decade, a great deal of attention has been focused on the favorable effects of antioxidants on the quality of cryopreserved boar sperm and fertilizing capacity, such as vitamin E [5,6], glutathione [7,8,9], l-cysteine [10,11], *N*-acetyl l-cysteine [3,12] and gamma-oryzanol [13,14]. In recent years, several plant-derived polyphenols such as quercetin (flavonoids) [15], resveratrol (non-flavonoids) [16], and curcumin [17] also revealed their potential antioxidant activities for boar sperm.

Resveratrol (trans-3,5,4′-trihydroxystillbene) is a natural polyphenolic compound found in grapes, mulberries, plums, peanuts, and other plant derived products [18]. Resveratrol exerts potent cytoprotective activity via its anti-inflammatory and antioxidant properties. As an effective antioxidant, resveratrol is capable of neutralizing free radicals such as hydroxyl, alkoxyl, and peroxyl radicals as well as reducing lipid peroxidation (LPO) [18,19]. Moreover, resveratrol indirectly increases intracellular antioxidant enzymes such as superoxide dismutase (SOD) and catalase [20]. Resveratrol has been extensively used as an antioxidant additive to semen extenders in human [21,22], ram [23], bull [24,25,26], buffalo [27], boar [16,28], and rooster [29] semen. It has been demonstrated that resveratrol minimizes DNA damage and protects the membrane integrity of human sperm after thawing procedure [21,22]. In addition, resveratrol has been shown to increase the sperm motility, mitochondrial activity, and DNA integrity of cryopreserved bull sperm [24,26]. In boar, Sun et al. [28] found that the addition of resveratrol to semen extender could protect boar sperm against oxidative stress and increase sperm survival during liquid storage. However, there is only one report about the effect of resveratrol on frozen boar semen [16]. Therefore, the purpose of this study was to determine the effect of resveratrol and its optimal concentration on the quality of post-thawed boar sperm.

## 2. Materials and Methods

### 2.1. Animals

Thirteen fertile Duroc boars (aged 1.5–3 years) utilized for routine artificial insemination in a commercial herd were used in this study. The boars were kept in an enclosed facility with an evaporative cooling system and separate enclosures. Each boar was fed a commercial diet containing 15–16% of protein twice daily, and water was provided ad libitum via water nipple.

### 2.2. Semen Collection

One ejaculate from each of the 13 individual boars was collected using the gloved-hand technique. During collection, sperm-rich fractions were collected after filtering through filter paper (Minitube, Tiefenbach, Germany) [30]. Within 30 min after collection, the concentration (×10^6^ sperm/mL) was measured using Photometer SDM1 (Minitube, Tiefenbach, Germany), subjective motility was analyzed at 400× using a light microscope, and sperm morphology was stained with William’s staining method and evaluated using a light microscope. Cryopreservation was only conducted on ejaculate that exhibited morphological normality and a motility rate of 80% [10,31].

### 2.3. Semen Freezing and Thawing Protocol

The conventional method of sperm freezing with liquid nitrogen was used to freeze all semen samples. After collection, the fresh semen was extended in a 1:1 (*v*/*v*) ratio with the Modena^TM^ extender (Swine Genetics International, Ltd., Cambridge, IA, USA). The extended semen was carefully transferred into 50 mL centrifuge tubes, equilibrated at 15 °C for 120 min, and then centrifuged at 15 °C at 800× *g* for 10 min (Hettich Rotanta 460R, Tuttlingen, Germany). After discarding the supernatant, the sperm pellet was re-suspended in a ratio of about 1-2:1 (*v*/*v*) with a lactose–egg yolk extender at a concentration of 1.5 × 10^9^ spermatozoa/mL. The extender consisted of 80 mL of an 11% lactose solution and 20 mL of egg yolk. In this phase, the sperm sample was divided into 7 groups based on the resveratrol (R5010, Sigma-Aldrich, Saint Louis, MO, USA) concentration (0, 25, 50, 75, 100, 125, and 250 µM in 0.5% dimethyl sulfoxide (DMSO)). All of the sperm samples were chilled to 5 °C for 90 min. Each sample group was mixed with freezing extender, which is composed of 89.5% lactose–egg yolk extender with 9% (*v*/*v*) glycerol and 1.5% (*v*/*v*) Equex-STM^®^ (Nova Chemical Sales Inc., Scituate, MA, USA), to a final concentration of 1.0 × 10^9^ sperm/mL [10,11]. Processed samples were placed into 0.5 mL plastic straws (IMV Technologies, L’Aigle, Basse-Normandie, France). The semen straws were frozen by contacting nitrogen vapor at 3 cm above the liquid nitrogen level for 20 min (−20 °C/min) in an expandable polystyrene box, then plunged into the liquid nitrogen tank (−196 °C) for storage prior to analysis [10]. All the frozen semen samples had been kept for 10 years before analysis. For sperm evaluation, two randomly selected frozen straw samples were thawed at 50 °C for 12 s and extended (1:4) with a pre-warmed Modena^TM^ extender at 37 °C for 15 min [10].

### 2.4. Assessment of Sperm Motility

Sperm motility was analyzed by computer-assisted sperm motility analysis (CASA) (AndroVision^®^, Minitube, Tiefenbach, Germany). Briefly, 3 µL of a semen sample was pipetted into a pre-warmed disposable counting chamber (Leja^®^ 20 µM, IMV Technologies, L’Aigle, Basse-Normandie, France) and maintained at 37 °C during analysis. At least 600 sperm cells were counted per analysis across five fields of each sample. The results of analysis were expressed as the percentage of total sperm motility and progressive motility as well as kinetic parameters such as curvilinear velocity (VCL, µm/s), average pathway velocity (VAP, µm/s), straight line velocity (VSL, µm/s), the amplitude of the lateral head displacement (ALH, µm), straightness (STR, %), and linearity (LIN, %). Motile spermatozoa were defined when VCL ≥ 24 μm/s and ALH > 1 μm. The term “progressive motility” (PMOT) has been interpreted as the presence of sperm exhibiting a VCL ≥ 48 μm/s and VSL < 10 μm/s [32]. The total motility (MOT) is the summation of sperm motility subpopulations that were determined by VCL thresholds, including local motility (VCL ≥ 24 and < 48 μm/s), slow motility (VCL ≥ 48 and < 80 μm/s), and fast motility (VCL ≥ 80 μm/s) [32].

### 2.5. Assessment of Sperm Viability

**Figure 1:**
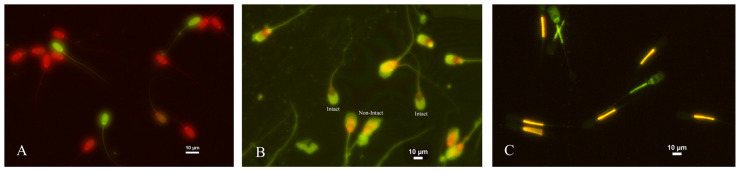
Assessment of frozen-thawed boar spermatozoa using specific fluorescent dyes (400× magnification). (**A**) Viable and non-viable sperm stained with SYBR-14 and EthD-1 (**B**) Intact and non-intact acrosomes assessed by FitC-PNA/EthD-1 staining (**C**) Mitochondrial membrane potential (MMP) stained with the dyes JC-1 and FitC-PNA; high MMP with orange fluorescence in midpiece; and low MMP with green fluorescence in midpiece.

Sperm viability was assessed using a staining technique of SYBR-14 (Sperm viability kit, Molecular probes, L7011) and Ethidiumhomodimer-1 (EthD-1). Briefly, 10 µL of semen was combined with 2.7 µL of SYBR-14 (10 µM in DMSO, final concentration of 0.54 µM) and 10 µL of 1.17 µM EthD-1. The mixture was incubated at 37 °C for 15 min. Under a fluorescence microscope with a magnification of 400×, 200 sperms were evaluated. The sperm stained with SYBR-14/EhtD-1 were separated into viable sperm and non-viable sperm. The nuclei of living sperm with an undamaged plasma membrane turned green, while the nuclei of damaged membranes or dead sperm turned red (Figure 1). The results were scored as the proportion of viable and non-viable sperm [10].

### 2.6. Assessment of Acrosome Integrity

The evaluation of acrosome integrity was conducted using a staining protocol of fluorescein isothiocyanate-labeled peanut agglutinin (FITC-PNA) and EthD-1 staining. A total of 10 µL of semen and 10 µL of EthD-1 were incubated at 37 °C for 15 min. Then, 5 µL of the mixture was applied to a glass slide and allowed to air dry. The slide was fixed with 95% ethanol for 30 s and then allowed to air dry. A total of 40 µL of FITC-PNA (100 µg/mL in PBS) was spread onto the slides and kept in a humid container at 4 °C for 30 min. The slides were then rinsed with cold PBS and air dried. At least 200 green-stained, live sperms were evaluated under a 1000× magnification fluorescence microscope and classified as intact acrosome or non-intact acrosome (Figure 1). The results were expressed as a percentage of intact and non-intact sperm acrosomes [10].

### 2.7. Assessment of Mitochondrial Membrane Potential

Mitochondrial membrane potential was assessed with a staining protocol of fluorochrome 5,5′,6,6′-tetrachloro-l,l′,3,3′-tetraethylbenzimidazolylcarbocyanine iodide (1.53 mM) (JC-1; T3168, Invitrogen, Waltham, MA, USA). A total of 50 µL of diluted semen was mixed with 3 µL of a 2.4 mM propidium iodide (PI) and 3 µL of a 1.53 mM JC-1 solution in DMSO and incubated for 10 min at 37 °C in a dark container. Under a fluorescence microscope with a magnification of 400×, two hundred sperm were evaluated. Midpiece staining revealed that sperm with a high mitochondrial membrane potential were yellow-orange, while sperm with a low membrane potential were green (Figure 1). The results were expressed as a percentage of sperm with high mitochondrial membrane potential [33,34].

### 2.8. Measurement of Malondialdehye (MDA) Levels

The MDA levels of semen samples were measured using the thiobarbituric acid reactive substance (TBARS) assay. Briefly, 250 µL of a semen sample was added with 250 µL of 0.2 mM ferrous sulphate and 250 µL of 1 mM ascorbic acid and incubated at 37 °C for 60 min. Thereafter, 1 mL of 15% (*w*/*v*) trichloroacetic acid was added to each tube, followed by 1 mL of 0.375% (*w*/*v*) thiobarbituric acid. The sample tubes were vortexed and boiled in a water bath for 10 min. Next, the samples were placed in an ice box to stop the reaction. Finally, the samples were centrifuged at 4 °C at 800× *g* for 10 min. The supernatant (2 mL) from each sample was transferred to a cuvette for analysis using a UV-visible spectrophotometer (SPECTRONIC^®^ 20 GENESYS^TM^; Spectrum Chemical Mfg. Corp., New Brunswick, NJ, USA). Absorbance was read at 532 nm. The levels of MDA were calculated from the MDA standard curve and expressed as µM/mL [35].

### 2.9. Statistical Analysis

Total motility, progressive motility, sperm kinetic parameters (VCL, VSL, VAP, ALH, STR and LIN), sperm viability, acrosome integrity, mitochondrial membrane potential, and level of MDA were analyzed using SPSS Statistics for Windows, version 26.0 (SPSS Inc., Chicago, IL, USA). The data were tested for normality and then analyzed with a one-way ANOVA followed by Duncan’s multiple range test to determine significant differences in all parameters. A statistically significant difference was defined as *p* < 0.05.

## 3. Results

### 3.1. Effect of Resveratrol on the Frozen-Thawed Boar Sperm Parameters

The descriptive statistics of the quality of fresh boar semen are presented in Table 1. The quality of fresh semen was acceptable by all measurements. The effect of the various concentrations of resveratrol on frozen-thawed boar sperm motility characteristics and other sperm parameters is presented in Table 2 and Table 3, respectively. The addition of 50–100 µM resveratrol enhanced total sperm motility, progressive motility, and sperm viability after thawing compared to the control group (*p* < 0.05). However, there was no significant difference in post-thawed sperm parameters among the 50, 75, and 100 µM resveratrol groups. For sperm kinetic parameters, the VCL, VSL, VAP, and ALH of the 50 µM resveratrol group were significantly higher when compared to the control group (*p* < 0.05). Nonetheless, the addition of resveratrol did not significantly affect the linearity and straightness of sperm movement. In this study, the highest total motility (49.2 ± 2.2%), sperm viability (52.2 ± 2.4%), and sperm with high mitochondrial membrane potential (60.7 ± 2.0%) were observed in the 50 µM resveratrol group. Nonetheless, the maximum concentration of resveratrol (250 µM) tended to decrease total sperm motility, sperm viability, intact acrosomes, and mitochondrial membrane potential. Furthermore, the 250 µM resveratrol group showed the lowest percentage of acrosome integrity.

### 3.2. Effect of Resveratrol on the Lipid Peroxidation

**Figure 2:**
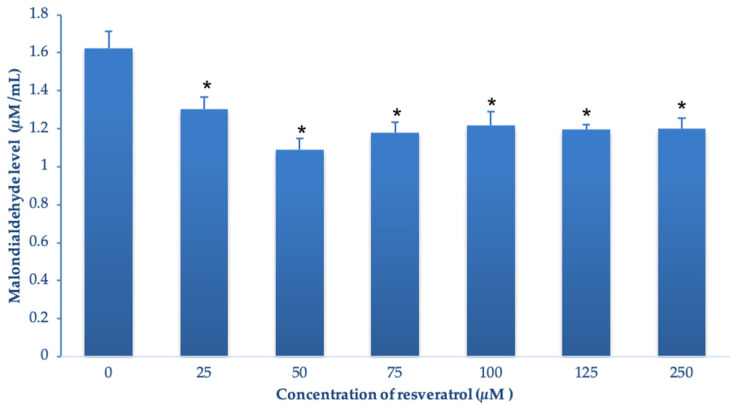
Malondialdehyde level of the frozen-thawed boar semen in different concentrations of resveratrol (7 groups). Asterisks indicate a statistically significant difference (*, *p* < 0.05).

The effects of resveratrol on lipid peroxidation during cryopreservation are shown in Figure 2. The highest level of MDA was observed in the control group (1.62 ± 0.1 µM/mL) compared with the resveratrol treatment groups (*p* < 0.05). The 50 µM resveratrol group had the lowest MDA level (1.08 ± 0.1 µM/mL). However, the level of MDA among treatment groups was not statistically significant.

## 4. Discussion

It is noteworthy that sperm cryopreservation induced excessive ROS generation that led to lipid peroxidation of the sperm plasma membrane as well as DNA oxidation. These events contribute to the impairment of sperm structure and function, the reduction in sperm survival rate, and the decrease in fertilizing capability [1,2]. In this study, resveratrol supplementation in freezing extender 50–100 µM protected the sperm from oxidative stress by reducing LPO and enhancing sperm motility, sperm viability, and high mitochondrial membrane potential following semen cryopreservation. Resveratrol has antioxidant properties via extracellular mechanisms that inhibit superoxide anion, scavenge free radicals, and donate protons as well as intracellular mechanisms that decrease lipid peroxidation of the sperm plasma membrane and protect sperm proteins, mitochondria, and DNA damage during the freezing and thawing process [22,36].

Our investigations are similar to those of other species, in that resveratrol was supplemented during the chilled storage and sperm cryopreservation processes. Bucak et al. [26] demonstrated that resveratrol at 1 mM increased the sperm motility and mitochondrial activity of post-thawed bull sperm. In buffalo sperm, the optimal concentration at 50 µM decreased capacitation-like change and oxidative stress, improving sperm membrane integrity and in vitro fertilizing capacity [27]. The addition of resveratrol at 50 µM and 150 µM to fresh semen extender significantly improved the quality of the boar sperm by minimizing ROS and MDA content during liquid storage and the fast cooling process, respectively [28]. Moreover, the 50 µM of resveratrol protected the quality of the boar sperm and reduced the LPO during cryopreservation [16]. In rooster semen, the study showed that 0.1 µM of resveratrol improved the sperm motility, membrane integrity, and mitochondrial activity [29]. Recently, resveratrol at 200 µM was the optimal concentration for improving the quality of cryopreserved dog semen, including sperm motility and survival rate [37]. In contrast to our findings, Garcez et al. [21] and Silva et al. [23] found that resveratrol supplementation did not enhance post-thawed sperm motility and mitochondrial activity in human and ram sperm. Resveratrol, on the other hand, could protect the DNA and plasma membrane of sperm after cryopreservation [21,23]. It has also been reported that 50 µM resveratrol protected against motility loss but not against oxidative damage induced by cryopreservation of ram sperm [20]. The difference in outcomes between the present study and prior studies might indicate that the protective effect of resveratrol on sperm quality not only depends on the concentration of resveratrol but also animal species, extender composition, storage conditions, and freezing and thawing methods utilized in earlier investigations [16,19,20,21,22,23,24,27,29,37,38] and reviewed by Chanapiwat and Kaeoket [39].

It is well documented that sperm cells need energy to conserve their metabolism and function [40]. It has been hypothesized that AMP-activated protein kinase (AMPK) is important in maintaining the balance of energy metabolism, and its involvement in sperm physiological function—specifically, sperm motility, plasma membrane integrity, and mitochondrial activity—has been demonstrated [41]. In boar spermatozoa, the AMPK protein is predominantly localized on the whole acrosome and midpiece of sperm [42]. During the freezing and thawing process, sperm undergo stressful conditions and subsequent fluctuations of sperm AMPK activity, which cause a rapid loss of sperm motility and a decrease in membrane fluidity, acrosome integrity, and mitochondrial activity [41]. As an AMPK activator, resveratrol is hypothesized to prevent sperm from cryodamage by activating AMPK phosphorylation [43]. Previous studies in rooster [29] and boar [16] sperm reported that the addition of resveratrol activated AMPK phosphorylation, reducing ROS generation and enhancing sperm antioxidative enzymes, which is correlated with a higher sperm progressive motility and mitochondrial function. This underlying mechanism may support the present results in that 50 µM resveratrol could enhance post-thawed boar sperm progressive motility, viability, intact acrosome, and mitochondrial activity. However, a decrease in sperm motility and intact acrosomes was observed in the 250 µM resveratrol group. Many studies have demonstrated that the concentration–effect relationships of resveratrol exhibit a biphasic pattern both in vitro and in vivo; specifically, it functions as an antioxidant at low concentrations and as a pro-oxidant at high concentrations [29,44,45]. Moreover, sperm has a limited capacity to uptake the antioxidants; too high of a concentration might not be beneficial [11,17]. In order to elucidate the cytotoxic effect, it has been reported that resveratrol at 100 µM displayed a cytotoxic effect on sperm motility and DNA damage of human sperm [22]. In addition, Bucci et al. [46] demonstrated that adding 2 mM resveratrol to a thawing medium had a negative impact on boar sperm motility and DNA integrity but did not influence sperm viability, mitochondrial function, or lipid peroxidation. Furthermore, a decrease in motility at the highest concentration (1 µM) was already observed in frozen-thawed rooster sperm [29], indicating variation in the cytotoxic level in different species. Hurtado de Llera et al. [42] demonstrated that activation of AMPK activity by an AMPK activator (A769662) caused a harmful effect on sperm motility and, in parallel, regulated the sperm plasma membrane, acrosome, and mitochondrial integrity of boar sperm. In terms of an AMPK activator, a too high concentration of resveratrol may interfere with the activation of AMPK phosphorylation, which is required for optimal sperm motility [41,42]. Nevertheless, additional research is required to examine the effects of resveratrol on fertilization and other reproductive performances in pig farms.

## 5. Conclusions

In this study, it is proven that resveratrol, a natural polyphenolic antioxidant, is capable of inhibiting lipid peroxidation and improving sperm motility, viability, intact acrosomes, and mitochondrial membrane potential in frozen-thawed boar sperm. The addition of 50–100 µM resveratrol to a freezing extender enhanced sperm motility and the viability of cryopreserved boar sperm. However, a detrimental effect of resveratrol was found at a concentration of 250 µM.

## Figures and Tables

**Table 1:** Descriptive statistics for sperm parameter measurements of fresh boar semen (*n* = 13).

Parameters	Mean ± SD	Range
Concentration (×10^6^ sperm/mL)	539.8 ± 155.4	451–825
Total motility (%)	83.0 ± 7.6	75–95
Sperm viability (%)	79.8 ± 8.1	75–90
Acrosome integrity (%)	91.6 ± 5.6	78–96
Sperm morphology (%)	88.3 ± 4.7	84–94

**Table 2:** Means ± SEM of sperm motility characteristics of frozen-thawed boar sperm among 7 groups of different resveratrol concentrations.

Parameters	Resveratrol Concentrations						
	Control	25 μM	50 μM	75 μM	100 μM	125 μM	250 μM
MOT	33.5 ± 3.3 ^b^	45.7 ± 2.5 ^ab^	49.2 ± 3.2 ^a^	48.9 ± 3.7 ^a^	48.7± 3.4 ^a^	47.6 ± 3.6 ^a^	36.4 ± 3.9 ^ab^
PMOT	23.8 ± 3.2 ^b^	37.3 ± 2.9 ^ab^	41.3 ± 3.0 ^a^	40.3 ± 3.8 ^a^	41.1± 3.6 ^a^	37.2 ± 3.9 ^ab^	25.9 ± 4.0 ^b^
VCL	31.8 ± 1.9 ^b^	38.1 ± 3.2 ^ab^	48.8 ± 2.6 ^a^	43.9 ± 2.9 ^a^	39.5 ± 2.9 ^ab^	38.3 ± 3.9 ^ab^	34.5 ± 2.5 ^b^
VSL	11.9 ± 0.9 ^b^	14.1 ± 1.3 ^ab^	16.5 ± 0.8 ^a^	15.7 ± 1.5 ^ab^	14.3 ± 1.4 ^ab^	11.9 ± 0.9 ^b^	13.4 ± 1.5 ^ab^
VAP	15.5 ± 1.1 ^b^	18.5 ± 1.6 ^ab^	24.5 ± 1.2 ^a^	22.1 ± 1.7 ^a^	18.1 ± 1.7 ^ab^	18.5 ± 1.6 ^ab^	17.6 ± 1.7 ^ab^
ALH	0.34 ± 0.03 ^b^	0.43 ± 0.04 ^ab^	0.46 ± 0.03 ^a^	0.41 ± 0.02 ^ab^	0.42 ± 0.03 ^ab^	0.43 ± 0.04 ^ab^	0.37 ± 0.04 ^ab^
STR	76.8 ± 1.7	76.1 ± 1.9	75.2 ± 1.2	77.7 ± 2.0	78.1 ± 1.5	76.8 ± 1.7	76.5 ± 1.4
LIN	37.5 ± 2.1	38.4 ± 1.9	36.8 ± 0.8	40.3 ± 2.0	38.7 ± 1.9	37.5 ± 2.1	39.0 ± 2.8

Values in each row marked with different superscript letters differ significantly (*p* < 0.05). MOT; total motility (%), PMOT; progressive motility (%), VCL; curvilinear velocity (μm/s), VSL; velocity straight line (μm/s), VAP; average pathway velocity (μm/s), ALH; amplitude of lateral head displacement (μm), STR; straightness (%), LIN; linearity (%).

**Table 3:** Means ± SEM of sperm viability (%), intact acrosome (%) and mitochondrial membrane potential (MMP; %) of frozen-thawed boar sperm among 7 groups (*n* = 13).

Group	Sperm Parameters		
	Viability	Intact Acrosome	MMP
Control	38.8 ± 4.0 ^b^	40.6 ± 2.5 ^b^	47.9 ± 1.5 ^c^
Resveratrol 25 μM	47.3 ± 1.9 ^ab^	50.0± 2.9 ^a^	55.9 ± 1.7 ^ab^
Resveratrol 50 μM	52.2 ± 2.4 ^a^	49.5 ± 1.8 ^a^	60.7 ± 2.0 ^a^
Resveratrol 75 μM	51.1 ± 3.4 ^a^	44.9 ± 3.4 ^ab^	58.0 ± 2.0 ^ab^
Resveratrol 100 μM	49.5 ± 2.4 ^a^	42.1± 2.9 ^ab^	57.7 ± 2.8 ^ab^
Resveratrol 125 μM	46.6 ± 2.7 ^ab^	43.7 ± 2.5 ^ab^	59.2 ± 2.5 ^ab^
Resveratrol 250 μM	44.5 ± 3.6 ^ab^	40.1 ± 2.1 ^b^	53.9 ± 2.5 ^bc^

Values in each column marked with different superscript letters differ significantly (*p* < 0.05).

## Data Availability

Not applicable.
